# Supplementary material for: Effects of Cholesterol Modulation on Cisplatin-Induced Hearing Loss
Source: bioRxiv. 2025 Sep 26:2025.09.23.678043. Preprint. [Version 1] doi: 10.1101/2025.09.23.678043 (PMC12485892; doi:10.1101/2025.09.23.678043)
Supplement: Supplement 1 — Supplementary Figure 1 – PCR-based genotyping followed by BsrI restriction digestion. A 310 bp band confirmed the deletion of Pcsk9, while the presence of a BsrI digestion product at 364 bp indicated successful replacement of the Cdh23ahl locus with the wild-type cast-derived Cdh23 allele. [file media-1.pdf]

Supplemental Information

Effects of Cholesterol Modulation on Cisplatin-Induced Hearing Loss

John Lee*, Marcello Peppi*, Megan Guidry, Katharine Fernandez, Abu Chowdhury,

Lizhen Wang, Lisa L. Cunningham

Regeneron - Internal

Supplementary Figure 1

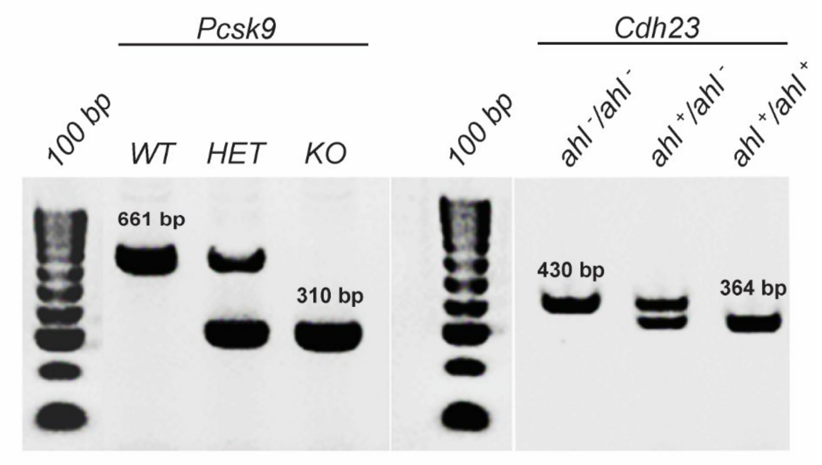

Figure 1 – PCR-based genotyping followed by BsrI restriction digestion. A 310 bp band confirmed the deletion of *Pcsk9*, while the presence of a BsrI digestion product at 364 bp indicated successful replacement of the *Cdh23*^{ahl} locus with the wild-type *cast*-derived *Cdh23* allele.

Regeneron - Internal
